# Patients’ health literacy in relation to the preference for a general practitioner as the source of health information

**DOI:** 10.1186/s12875-019-0975-y

**Published:** 2019-07-06

**Authors:** Monika Oedekoven, Wolfram J. Herrmann, Clemens Ernsting, Susanne Schnitzer, Melanie Kanzler, Adelheid Kuhlmey, Paul Gellert

**Affiliations:** 1 Institute of Medical Sociology and Rehabilitation Science; Charité – Universitätsmedizin Berlin, Berlin, Germany; 2 Institute of General Practice, Charité – Universitätsmedizin Berlin, Berlin, Germany; 3 Furtwangen University, Furtwangen, Germany; 4 Deutscher Evangelischer Krankenhausverband e.V, Berlin, Germany

**Keywords:** General practitioner, Health literacy, Action planning, Self-efficacy, Source of health information

## Abstract

**Background:**

For many patients, the general practitioner (GP) is the most important point of contact for obtaining information about a wide range of health topics. However, patients with different characteristics may seek health information from different sources, such as friends or the internet. The relationship between patient characteristics and preferences for information sources is understudied. We investigate which information sources are used by patients for health-related questions and how this relates to patients’ sociodemographics, health, and health literacy.

**Methods:**

A stratified and population-based survey was conducted to investigate health information sources within the German population over 35 years (*n* = 4144). Sociodemographics, use of technology, health-related indicators, and health literacy (including self-efficacy and action planning), as well as questions regarding the ratings of multiple health-related information sources, were investigated in personal interviews and analyzed using logistic regression.

**Results:**

In our study, GPs were the most important source of information for the patients, followed by medical specialists, pharmacists and the internet. Patient age and number of illnesses were associated with the choice of information source. Furthermore, action planning and self-efficacy for acquiring health knowledge were associated with the selected source of information.

**Conclusions:**

Information provider appears to be an important role for GPs, particularly among old and chronically ill patients. GPs should have the specific capabilities to fill this role and should be trained and referred to accordingly. Self-efficacy and action planning for acquiring health knowledge are important patient factors doctors can use for brief inventions during consultations.

## Background

For patients, selecting appropriate sources of health information and understanding the information provided are crucial for optimal health outcomes [1]. For physicians, patient-centered care requires an understanding of patients’ sources of health-related information. Such knowledge can inform healthcare professionals’ efforts to develop effective interventions and strategies to help patients and their family caregivers obtain high-quality health information and participate in healthcare decisions about themselves and their loved ones [1].

Navigating the healthcare system is challenging, and patients’ health literacy appears to be critical for understanding health information [2]. Health literacy is the ability to obtain, read, understand, and use information to make appropriate health decisions and follow instructions for treatment [3]. Past studies have determined that low health literacy is associated with limited understanding of verbal and written medical instruction, limited knowledge of healthcare services, a high risk of hospitalization, high mortality, decreased probability of screening and prevention, and lower levels of health behavior and treatment adherence [4]. People with low levels of health literacy often suffer from chronic diseases or are more likely to be disabled [5]. Although the majority of the German population stays actively informed about health topics, a representative survey shows that approximately 54% of the population has limited health literacy [6].

Furthermore, health literacy is firmly connected to concepts such as self-efficacy and action planning in the context of health and health behaviors [7, 8]. Self-efficacy is “the belief in one’s capabilities to organize and execute the courses of action required to manage prospective situation [9].” While these concepts can correlate with health literacy, they can also be seen as facets of health literacy. In addition, previous findings have revealed that health literacy interventions improve health outcomes such as self-efficacy [10–12].

The question arises whether inadequate health literacy relates to poor choices or the availability of sources of health information. Commonly mentioned sources of health information are doctors or other healthcare professionals, acquaintances and friends, and mass media, such television, radio, and newspapers [13–15]. More recently, internet and health apps have gained importance as sources of health information [16, 17]. Although the internet is utilized by many individuals, studies show that the most common and trusted source of information is healthcare professionals [18, 19], although the ranking varies across studies [5, 19, 20].

In addition to sociodemographic (e.g., age, gender) and health-related (e.g., health status) patient characteristics, a patient’s choice of a certain source of health information may be associated with his or her health literacy and confidence in information seeking [21–25]. Among all age groups, individual consultation with a doctor is rated more important than the search for information in the internet [26]. However, with increasing age, this importance of the general practitioner (GP) as a source of information becomes even stronger (i.e., it shows a linearly increasing trend) [19, 27]. Conversely, younger individuals use the internet more intensively (i.e., GP consultation shows a decreasing trend) [19]. Other studies suggest that health literacy is low in younger cohorts, increases in middle- and young-old cohorts and decreases again among the oldest old, i.e., a quadratic or curvilinear trend across age cohorts has been observed [28]. Furthermore, previous studies confirm a gender gap in health information-seeking behavior [29–32]. In general, women were found to be more engaged in seeking health-related information through the internet than men [32]. Health-related factors were found to be associated with the choice of information source. For example, people with better health were more likely to seek health information on the internet [33]. In addition, cancer patients who were in poor health preferred to seek a doctor or a healthcare provider for health information rather than other sources [34]. Moreover, among cancer survivors, those with lower educational attainment used healthcare providers as a source of information less frequently than those who were highly educated [35]. Several studies indicate that a person’s use of health information sources is related to his or her health literacy [2, 15, 17, 18, 24, 36]. Until now, the concepts of health literacy, planning and self-efficacy have not been examined in combination with the choice of health information source.

To date, studies have focused largely on online sources of health information. Only a limited number of studies have explored the lack of evidence concerning the associations among sociodemographic, health-related factors, health literacy and the use of offline information sources. In particular, the correlation between health beliefs/health literacy (such as self-efficacy and action planning) and information sources has not been previously examined. Thus, the aims of this study were to (a) investigate the proportion of use of the most common health-related sources of information among the general population and to (b) identify sociodemographics and health-related correlates (c) and determine health literacy (including self-efficacy and action planning) in relation to these sources of information. Concerning age trends (sociodemographics, b) specifically, we expected to find a nonlinear pattern that changes from young to middle to old and to very old age for all information sources.

## Methods

### Sampling and procedure

Data from the Monitor Survey, a stratified, population-based sample of 4144 individuals from Germany aged 35 years and older, was used in this cross-sectional study. The participants were recruited by the interviewers either door-to-door, in public places or at work. The criteria for participation were as follows: a permanent residence in Germany, adequate language skills and a minimum age of 35 years. Furthermore, the sample was stratified by age, gender, education level and German federal state to ensure the current sample is representative [37]. The response rate was 55%. Finally, the participants were interviewed by computer-assisted personal interviews at their homes in July 2015. Of the interviewed individuals, 7% failed to complete the survey, and their data were subsequently deleted. Those dropouts were excluded from the final sample (*N* = 4144). The mean amount of time the participants needed to finish the survey was 29 min. All participants gave written informed consent for participation in the study. The questionnaire was conducted in compliance with the Declaration of Helsinki. After a telephone consultation, the present analysis of anonymous data was classified as secondary data analysis by the head of the local ethics committee office (Ethics committee; Charité – Universitätsmedizin Berlin, Berlin, Germany) without need of further evaluation. In Germany, secondary data analyses do not require ethical approval [38]. All data were collected and analyzed anonymously. Therefore, ethical approval was not obtained.

### Measures

### Primary endpoint

The sources of information regarding health and illness were evaluated using a questionnaire that was based on evidence-based practice. The following categories of information sources were presented: magazines, pharmacies, the internet, health insurance, patient support groups, general practitioner, specialist, doctor’s assistant and nurse, friends/acquaintances, and other information sources. There was also an option for those who were not informed at all: “I do not actively seek information about illnesses and medical questions”. Multiple answers were possible.

### Sociodemographics

Age, gender, education (International Standard Classification of Education: ISCED), occupation, income and first language were assessed by standard survey items. In addition, the participants were asked whether they owned and used an internet-capable smartphone. Post-tax household income by month was categorized as follows: low < 2100 Euros; moderate 2100–3600 Euros; high > 3600 Euros (1 Euro = 1.13 US Dollar [June 07, 2019]).

### Chronic conditions, health (related) behavior, health-related quality of life

Chronic conditions were assessed by asking participants the following question: Do you suffer from one or more of the following chronic conditions: a) cardiovascular disease, b) cancer, c) lung and respiratory diseases, d) musculoskeletal system conditions, e) major depression, f) chronic pain, g) diabetes mellitus, h) hypertension, i) other chronic conditions?" The responses were subsequently categorized as “none”, “one”, “two”, and “three or more”. Furthermore, the body mass index (BMI) was calculated using self-reported weight and height (BMI = weight (in kg)/ height (in squared m)). Health-related behavior (smoking, physical activity, balanced diet) was assessed dichotomously (0 = no, 1 = yes). For smoking, the participants were asked “Do you smoke on a daily basis?” To assess *physical activity*, the participants were asked “Are you regularly physically active (following WHO recommendations, i.e., 5 times per week 30 min of moderate activity 5 times per week or 30 min of intensive activity 3 times per week [15])?” *Balanced diet* was measured by asking the participants “Do you follow a balanced diet, i.e., eat fruits and vegetables with every meal and consume many wholegrain products?” Health-related quality of life was measured using the EUROHIS-QOL 8-Item Index, which had a Cronbach’s alpha of 0.90 in the current analysis [39]. Example items included “How would you rate your quality of life?” and “How satisfied are you with your health?” All items were answered on a 5-point Likert scale.

### Health literacy

*Perceived health literacy* was measured using the HLS-EU-Q [3]. This validated instrument consists of 16 items and had a Cronbach’s alpha of 0.87 in the present study. Scores range from 0 to 50 and reflect the individual’s perceived capability to acquire, understand and act on health information. An example item is “On a scale of very simple to very difficult, how easy is it to understand what the doctor tells you?” Answers had a 4-point response format on a Likert scale.

*Health knowledge* was questioned using a validated knowledge test with 36 statements with a Cronbach’s alpha of 0.73, which could be correct or false [40]. The test statements refer to knowledge of cardiovascular disease, cancer, lung and respiratory diseases, musculoskeletal system conditions, major depression and chronic pain.

*Self-efficacy* and *action planning* for acquiring health knowledge were adopted from the context of health behavior change and specified in regards to the acquisition of health knowledge in the present study [41]. An example item for self-efficacy is “I am sure that I can improve my knowledge of health”; and example item for action planning is “I have already precisely planned how to generate health knowledge.” All answers were given on a 5-point Likert scale.

### Statistical analysis

For the most important information source (i.e., GP), binary logistic regression models (i.e., GP as source yes/no) were used to test the hypotheses. Age (linear, square and cubic trends), gender and educational level, smartphone usage, health-related characteristics and health literacy (perceived health literacy [HLS-Q16], health knowledge, action planning and self-efficacy to acquire health knowledge) were included as covariates in the analyses. While higher-order trends (i.e., cubic and quadratic) were expected a priori for all sources of information, insignificant higher-order trends were subsequently removed from the final parsimonious models [42]. The statistical analyses were conducted using SPSS v25 statistical software.

## Results

In our study, 51.0% of the 4144 participants between the ages of 35 and 92 years (M = 56.9; SD = 13.5) were men, 12.9% did not have a basic education, and 18.1% held a university degree. The majority of the sample (58.3%) reported no chronic conditions, while 30.8% reported having one chronic condition, 11.2% reported having two, and 4.1% reported having three or more. Hypertension (18.4%), musculoskeletal conditions (9.3%) and cardiovascular diseases (9.1%) were the most commonly reported conditions. The mean BMI was 24.9 (SD = 3.5). Regarding health behavior, 28.5% of the participants claimed to smoke on a daily basis, 38.9% reported being physically active, and 60.9% consumed a balanced diet.

### Information sources

**Fig. 1:**
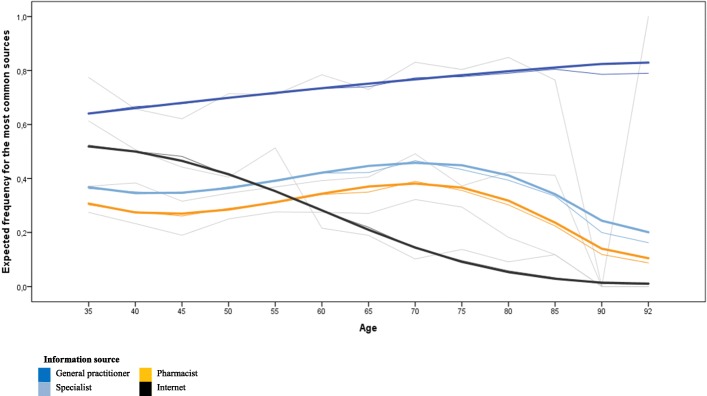
Expected frequency of stating general practitioner as information source (dark blue), specialist (light blue), pharmacist (orange) as well as internet (dark grey) by age. Bold colored lines represent unadjusted linear, quadratic or cubic age trend models (models 1). Thin colored lines represent adjusted models for age, gender, education, German as first language, smartphone use, amount of diseases, health-related behavior, BMI, health-related quality of life, health literacy and health knowledge as well as action planning and self-efficacy (models 2). Thin light grey lines reflect the averaged answers of the participants of the according age group

A total of 72.1% of the participants stated that the GP was their information source of choice for health-related questions (Table 1), followed by medical specialists (39.5%), pharmacists (31.6%), and the internet (31.5%). In all, 12.5% of participants claimed to not actively search for health information. The choice of the GP as an information source was linearly associated with age (Table 2 and Fig. 1 for the four most common sources; further details in the Appendix). Gender, education, first language, and app usage showed no relation to the preference of the GP as an information source. The relations between age and GP preference were consistent in the further-adjusted model (Table 3). Smokers (OR: 0.76 [0.65; 0.89]; *p* < 0.01; β = − 0.27) and participants with a lower quality of life (OR: 0.78 [0.67; 0.90]; *p* < 0.05; β = − 0.25) had a decreased probability of preferring the GP as an information source (Table 3). Participants who were suffering from multiple chronicle conditions preferred the GP as an information source compared with those without chronic conditions (OR: 1.16 [1.03; 1.3]; *p* < 0.05; β = 0.15). Perceived health literacy (HLS-Q16) and health knowledge were not significantly associated with the preference for GPs as a source of health information. Action planning (OR: 1.20 [1.12; 1.28]; *p* < 0.001; β = 0.18) and self-efficacy (OR: 1.50 [1.38; 1.6]; *p* < 0.001; β = 0.40) for acquiring health information had health-enhancing connotations in regards to the GP as an information source, superseding sociodemographic and health indicators (Table 3).

**Table 1:** Sample characteristics *(N = 4144)*

Socio-demographic description *n (%)*		Health-related description *n (%)*	
Gender (male)	2112 (51.0)	Multiple chronic conditions	
Age in years	M = 56.9 (SE = 13.5)	None	2231 (53.8)
		One	1278 (30.8)
Educational level (ISCED)		Two	466 (11.2)
No or basic education	534 (12.9)	Three or more	169 (4.1)
Vocational education	2858 (69.0)		
University degree	752 (18.1)	Chronic conditions	
		Cardiovascular disease	376 (9.1)
Occupational status		Cancer	79 (1.9)
Working full-time	2224 (53.7)	Respiratory diseases	232 (5.6)
Working part-time	434 (10.5)	Musculoskeletal system conditions	385 (9.3)
Not working	198 (4.8)	Depression	128 (3.1)
Retired	1287 (31.1)	Chronic pain	310 (7.5)
In school	1 (0.0)	Diabetes	361 (8.7)
		Hypertension	763 (18.4)
Monthly post tax household income			
Low	2165 (52.2)	BMI	M = 24.9 (*SE* = 3.5)
Medium	1107 (26.7)	Health behaviors	
High	290 (7.0)	Smoking	1181 (28.5)
No answer	582 (14.0)	Physical activity	1614 (38.9)
		Healthy diet	2522 (60.9)
First language			
German	3773 (91.0)		
Other	371 (9.0)	Health-related quality of life	M = 3.9 (*SE* = 0.6)
Information source			
General practitioner	2989 (72.1)	Health Literacy	
Specialist	1635 (39.5)	Perceived health literacy	M = 33.5 (SE = 7.4)
Pharmacist	1310 (31.6)	Health knowledge	M = 65.5 (SE = 17.3)
Im Internet	1305 (31.5)	Action planning	M = 3.04 (SE = 1.22)
Friends/ Acquaintance	1044 (25.2)	Self-efficacy	M = 3.7 (SE = 1.04)
Magazines	942 (22.7)		
Health insurance	591 (14.3)		
Do not actively search for health information	517 (12.5)		
Doctor’s assistant/ Nurse	279 (6.7)		
Other information sources	164 (4.0)		
Patient support groups	128 (3.1)		

*Note. M* Mean. *SE* Standard deviation. *ISCED* International Standard Classification of Education

**Table 2:** *Sources of information regarding health topics stratified by age and gender*

	All age group		35–44 years		45–54 years		55–64 years		65–74 years		75+ years		Unadjusted models ^b^	
	*n* = 2032 (%)	*n* = 2112 (%)	*n* = 450 (%)^c^	*n* = 461 (%)^c^	*n* = 524 (%)^c^	*n* = 542 (%)^c^	*n* = 438 (%)^c^	*n* = 450 (%)^c^	*n* = 363 (%)^c^	*n* = 388 (%)^c^	*n* = 257 (%)^c^	*n* = 271 (%)^c^	OR	
Variable ^a^	*M*	*F*	*M*	*F*	*M*	*F*	*M*	*F*	*M*	*F*	*M*	*F*	*Aging trend*	*M*
General practitioner	71.0 (1442)	73.2 (1547)	65.1 (293)	69.8 (322)	64.5 (338)	69.9 (379)	74.4 (326)	75.3 (339)	75.5 (274)	74.5 (289)	82.1 (211)	80.4 (218)	1.02*** (linear trend)	0.90
Specialist	39.0 (793)	39.9 (842)	34.9 (157)	36.7 (169)	33.4 (175)	35.8 (194)	43.2 (189)	44.0 (198)	43.5 (158)	44.6 (173)	44.4 (114)	39.9 (108)	1.00*** (cubic trend)	0.96
Pharmacist	31.3 (636)	31.9 (674)	28.2 (127)	28.4 (131)	28.2 (148)	28.4 (154)	34.5 (151)	34.2 (154)	34.7 (126)	37.9 (147)	32.7 (84)	32.5 (88)	1.00*** (cubic trend)	0.97
Internet	33.7 (684)	29.4 (621)	50.7 (228)	48.4 (223)	42.9 (225)	39.1 (212)	33.8 (148)	28.4 (128)	17.9 (65)	10.3 (40)	7.0 (18)	6.6 (18)	0.99*** (square trend)	1.23***
Friends/ Acquaintance	24.0 (488)	26.3 (556)	20.0 (90)	28.0 (129)	23.5 (123)	24.5 (133)	26.9 (118)	28.2 (127)	26.4 (96)	22.9 (89)	23.7 (61)	28.8 (78)	1.00 (linear trend)	0.89
Magazines	20.5 (416)	24.9 (526)	18.0 (81)	24.5 (113)	16.6 (87)	24.0 (130)	24.7 (108)	25.8 (116)	23.1 (84)	26.0 (101)	21.8 (56)	24.4 (66)	1.01 (linear trend)	0.78***
Health insurance	14.2 (289)	14.3 (302)	13.3 (60)	16.5 (76)	14.5 (76)	14.9 (81)	18.0 (79)	16.9 (76)	12.4 (45)	12.4 (12,4)	11.3 (29)	7.7 (21)	0.99** (square trend)	0.99
I do not actively search for information	13.2 (268)	11.8 (249)	14.9 (67)	12.4 (57)	16.8 (88)	14.0 (76)	11.9 (52)	10.7 (48)	9.9 (36)	11.6 (45)	9.7 (25)	8.5 (23)	1.00 (linear trend)	0.86
Doctor’s assistant/ Nurse	6.6 (135)	6.8 (144)	3.8 (17)	4.3 (20)	5.9 (31)	5.2 (28)	6.4 (28)	5.3 (24)	10.2 (37)	10.3 (40)	8.6 (22)	11.8 (32)	1.03*** (linear trend)	0.98
Other information sources	3.8 (78)	4.1 (86)	5.3 (24)	4.6 (21)	2.9 (15)	4.2 (23)	3.7 (16)	4.0 (18)	3.3 (12)	3.4 (13)	4.3 (11)	4.1 (11)	1.00 (linear trend)	0.94
Patient support groups	2.9 (58)	3.3 (70)	2.2 (10)	3.5 (16)	2.7 (14)	3.1 (17)	3.0 (13)	3.1 (14)	4.1 (15)	4.6 (18)	2.3 (6)	1.8 (5)	0.99 (linear trend)	1.14

*Note.* ^a^ Column is reflecting research question (a), i.e., the most important information sources. ^b^ Columns reflect research question (b), i.e., the information sources by age (linear, square, cubic trend) and gender, adjusted models additionally included education as well as amount of chronic diseases as covariate (not displayed). ^c^ % of participants of this specific gender and age group who named this source of information. *M* male, *F* female. *OR* Odds Ratio. ** *p* < .01; ***p < .001

**Table 3:** *Multivariate associations of the four most important information sources by socio-demographic and health-related indicators*

	General practitioner	
	Model 1	Model 2
Variable	unadjusted	adjusted
	*OR (LL 95%CI- UL95%CI)*	*OR (LL 95%CI- UL95%CI*
Intercept	2.77*** (1.01–1.02)	0.99*** (1.01–1.02)
Socio-demographic factors ^a^		
Age		
Linear	1.02***	1.02**
Square	**–**	**–**
Cubic	**–**	**–**
Gender (male)	0.90 (0.78–1.02)	0.93 (0.80–1.07)
Education (vocational education)		0.86 (0.71–1.04)
Education (university degree)		0.91 (0.69–1.17)
First language ^a^		0.90 (0.6–1.16)
Technological factors ^a^		
Smartphone Use		1.19 (0.98–1.44)
Health-related factors ^a^		
Multiple chronic conditions		1.16** (1.03–1.30)
BMI		1.00 (0.97–1.01)
Smoking		0.76** (0.65–0.98)
Healthy diet		1.11 (0.94–1.29)
Physical activity		1.05 (0.90–1.23)
Health-related quality of life		0.78* (0.67–0.90)
Health literacy ^b^		
Perceived health literacy (HLS-Q16)		1.01 (0.99–1.01)
Health knowledge		1.00 (0.99–1.00)
Action planning to acquire health knowledge		1.20*** (1.12–1.28)
Self-efficacy to acquire health knowledge		1.50*** (1.39–1.61)
Nagelkerke R^2^	0.02	0.11

*Note.* ^a^ Listed variables are  reflecting research question (b) (associated socio-demographic and health-related factors of information source) ^b^ Listed variables are reflecting research question (c) (associated health literacy factors of information source)

*OR* Odds Ratio, LL 95%CI- UL 95%CI = Lower limit and upper limit 95% confidence interval. *p < .05; ** *p* < .01; ***p < .001

## Discussion

In the present survey, GPs constituted the most important source of information, especially among older and chronically ill participants. Medical specialists, pharmacists and the internet were also important information sources. Finally, self-efficacy and action planning to acquire health information, but not perceived health literacy, were related to the preference for the GP as an information source.

Our analyses showed that GPs, followed by medical specialists, pharmacists and the internet, were the most important information sources (research question a). Although the internet and health apps have become increasingly popular [16], in line with our findings, previous studies show that GPs remain the most important information source [13–15]. In contrast to research based on samples consisting primarily of internet users, previous work has shown that personal communication and exchanges with doctors are still significantly more important than the internet in adult populations [43].

Concerning sociodemographic factors associated with source of information choice (research question b), in our study, higher age correlated linearly with a higher likelihood of choosing the GP as a source of information, which was found previously [27], although all age groups showed high levels of preference for the GP as an information source [19, 26, 27]. As expected, the internet was more frequently mentioned by younger age groups, which has also been found in other studies [19]. The preference for medical specialists and pharmacists showed a curve-shaped trajectory, with younger and very old participants showing lower values; this finding is in accordance with some findings on health literacy across age cohorts [28] but needs further replication in future studies. With regard to health-related characteristics (research question b) that were associated with the preference for GPs as an information source, the likelihood of choosing a GP increased as patients reported more chronic conditions and lower the quality of life. Following the results of an American survey, poor health status was correlated with the use of the GP as a source of information [33]. Furthermore, another American national survey showed that the proportion of chronically ill people who received personal information from a doctor was higher than the proportion of healthier people. In this survey, the proportion of chronic conditions was positively associated with the acquisition of information through a hospital doctor [44, 45]. Despite this finding, the internet still represents an important source of information for chronically ill people and is often rated positively by its users. More chronically ill patients than healthy people reported that they would still anticipate talking with a doctor in person following an online search [45, 46]. In the present survey, smokers were less likely to state the GP as an information source. Previous studies showed that although family and friends are a common source of information when quitting smoking, 70% of smokers consult a GP [47, 48]. A short targeted discussion of smoking problems can initiate an intervention for smoking cessation and increase the likelihood of quitting by twofold [49].

We did not find perceived health literacy or health knowledge to be consistently related to the choice of health information sources (research question c), which contradicts a substantial body of literature on the relationship between health literacy and information source [2, 15, 17, 18, 24, 36]. Nonetheless, self-efficacy and action planning were significantly related to the preference for GPs, medical specialists, and pharmacists as information sources, which adds to the health literacy literature and highlights the fact that concepts that are more specific to the acquisition of health information, such as self-efficacy and action planning, have more predictive value than more general concepts, such as perceived health literacy.

### Strengths and limitations

A strength of the present study is the broad nationwide sample. Although preferences regarding information sources for health-related topics is quite well described in the existing literature, our study could highlight the relevant characteristics that are related to these choices. In particular, the value of health- and health literacy-related characteristics is an important new finding. Limitations include the cross-selection design. A further limitation is the fact that, even though the preferred information source was clear, the frequency or intensiveness of its use remains unknown.

## Conclusions

Our results showed that GPs are the preferred source of information for the general population, especially older and chronically ill people. Furthermore, we showed that action planning and self-efficacy are positively related to the choice of GP as an information source. From our results, we can conclude that health-related information brokerage is an important physician task. GPs should have the relevant competencies and should be promoted and trained accordingly. Self-efficacy and action planning are important aspects that can be utilized for brief interventions during doctor-patient consultations. This competency is described as *communicator* in the CanMEDS Roles and can be found in the international standards for further education for GPs [50].

Although the teaching of communication competences at medical universities has increased in Germany, this aspect should also be emphasized in continuing education for practicing doctors. A targeted discussion initiated through the doctor can increase patients’ health-enhancing behaviors. An example includes motivational interviewing techniques, which can be utilized during patient talks [7]. The present study showed that self-efficacy and action planning play a substantial role in the medical context. Action plans created together with the patient that specify when, where, how, and with whom the desired information should be gathered, evaluated and used should be used in GPs’ practice. Previous studies have shown that even one-minute planning interventions can positively influence implementation [8, 51]. Especially in overloaded doctor offices, specific, brief action planning pays off. Increasing patients’ self-efficacy to verbalize or take notes and engage in specific brief action planning can be time-efficient strategies for the doctor’s practice as well as serving as health-improving and preventative strategies for patients [7]. Finally, brief interventions that promote physical activity in primary care and the community are more cost-effective than the usual care [52].

## Data Availability

The main results of the Monitor Survey are publicly available on the following webpages: https://www.monitor-versorgungsforschung.de/news/gut-aber-ausbaufaehig-so-viel-wissen-die-deutschen-ueber-gesundheit/image/image_view_fullscreen or https://www.charite.de/service/pressemitteilung/artikel/detail/wie_viel_wissen_die_deutschen_ueber_gesundheit/ . Further access to the data is available at request at the authors Paul Gellert (paul.gellert@charite.de) and Monika Oedekoven (monika.oedekoven@charite.de).

## Appendix

**Table 4:** Multivariate Associations of the four most important information sources by socio-demographic and health-related indicators

	General practitioner		Medical specialist		Pharmacist		Internet	
	Model 1	Model 2	Model 1	Model 2	Model 1	Model 2	Model 1	Model 2
Variable	unadjusted	adjusted	unadjusted	adjusted	unadjusted	adjusted	unadjusted	adjusted
	*OR*	*OR*	*OR*	*OR*	*OR*	*OR*	*OR*	*OR*
Axis intercept	**2.77*****	**0.99*****	**0.69*****	**0.03*****	**0.49*****	**0.01*****	**0.44*****	**0.02*****
Sociodemographic								
Age								
Linear	**1.02*****	**1.02****	**1.03*****	**1.02****	**1.03*****	1.02**	**0.94*****	**0.97*****
Square	**–**	**–**	**1.00**	**1.00**	1.00	**1.00**	**0.99*****	**0.99****
Cubic	**–**	**–**	**1.00*****	**1.00***	**1.00*****	**1.00****	**–**	**–**
Gender (male)	0.90	0.93	0.96	0.99	0.97	0.92	**1.23****	**1.17***
Education (vocational education)		0.86		**0.84***		**0.74****		1.20
Education (university degree)		0.91		1.17		**1.30***		0.76
First language		0.90		0.85		1.16		0.95
Technological								
Smartphone Use		1.19		1.15		**0.77****		**3.14*****
Health-related								
Multiple chronic conditions		**1.16****		**1.44*****		**1.42*****		**0.74*****
BMI		1.00		0.99		**1.04*****		1.02
Smoking		**0.76****		1.01		0.91		1.12
Healthy diet		1.11		**1.56*****		**1.26****		1.14
Physical activity		1.05		**1.24****		0.89		1.14
Health-related quality of life		**0.78***		0.99		**1.44*****		0.90
Health literacy								
Perceived health literacy (HLS16)		1.01		**1.01***		**1.02*****		**1.03*****
Health knowledge		1.00		1.01		**0.99****		**1.01*****
Action planning to acquire HK		**1.20*****		**1.23*****		**1.20*****		1.06
Self-efficacy to acquire HK		**1.50*****		**1.37*****		**1.24*****		1.05
Nagelkerke R^2^	0.02	0.11	0.01	0.13	0.01	0.11	0.17	0.25

*Note. OR* Odds Ratio, *HK* Health knowledge. **p* < .05; ** *p* < .01; ****p* < .001

## Abbreviations

*BMI*: Body mass index
*CAPI*: Computer-assisted personal interviews
*EUROHIS-QO*: *European Health* Interview *Survey-Quality* of *Life*
*GP*: General practitioner
*HLS-EU-Q*: European Health Literacy Survey Questionnaire
*HLS-Q16*: European Health Literacy Survey Questionnaire (16 Items)
*ISCED*: International Standard Classification of Education
*M*: Mean
*OR*: Odds ratio
*SD*: Standard deviation
*WHO*: World Health Organization
